# Predominance of multidrug-resistant bacteria causing urinary tract infections among symptomatic patients in East Africa: a call for action

**DOI:** 10.1093/jacamr/dlae019

**Published:** 2024-02-14

**Authors:** Antonio Maldonado-Barragán, Stephen E Mshana, Katherine Keenan, Xuejia Ke, Stephen H Gillespie, John Stelling, John Maina, Joel Bazira, Ivan Muhwezi, Martha F Mushi, Dominique L Green, Mike Kesby, Andy G Lynch, Wilber Sabiiti, Derek J Sloan, Alison Sandeman, John Kiiru, Benon Asiimwe, Matthew T G Holden

**Affiliations:** School of Medicine, University of St Andrews, St Andrews, Fife KY16 9TF, UK; Department of Microbiology and Immunology, Weill Bugando School of Medicine, Catholic University of Health and Allied Sciences, Mwanza P.O. Box 1464, Tanzania; School of Geography and Sustainable Development, University of St Andrews, St Andrews, Fife KY16 8AL, UK; School of Biology, University of St Andrews, St Andrews, Fife KY16 9TH, UK; School of Medicine, University of St Andrews, St Andrews, Fife KY16 9TF, UK; Department of Medicine, Brigham and Women's Hospital and Harvard Medical School, Boston, MA, USA; Centre for Microbiology Research, Kenya Medical Research Institute, Nairobi, Kenya; Department of Microbiology and Immunology, Mbarara University of Science and Technology, Mbarara, Uganda; Department of Microbiology and Immunology, Mbarara University of Science and Technology, Mbarara, Uganda; Department of Microbiology and Immunology, Weill Bugando School of Medicine, Catholic University of Health and Allied Sciences, Mwanza P.O. Box 1464, Tanzania; School of Geography and Sustainable Development, University of St Andrews, St Andrews, Fife KY16 8AL, UK; School of Geography and Sustainable Development, University of St Andrews, St Andrews, Fife KY16 8AL, UK; School of Medicine, University of St Andrews, St Andrews, Fife KY16 9TF, UK; School of Medicine, University of St Andrews, St Andrews, Fife KY16 9TF, UK; School of Medicine, University of St Andrews, St Andrews, Fife KY16 9TF, UK; School of Medicine, University of St Andrews, St Andrews, Fife KY16 9TF, UK; Centre for Microbiology Research, Kenya Medical Research Institute, Nairobi, Kenya; Department of Medical Microbiology, College of Health Sciences, Makerere University, Kampala, Uganda; School of Medicine, University of St Andrews, St Andrews, Fife KY16 9TF, UK

## Abstract

**Background:**

In low- and middle-income countries, antibiotics are often prescribed for patients with symptoms of urinary tract infections (UTIs) without microbiological confirmation. Inappropriate antibiotic use can contribute to antimicrobial resistance (AMR) and the selection of MDR bacteria. Data on antibiotic susceptibility of cultured bacteria are important in drafting empirical treatment guidelines and monitoring resistance trends, which can prevent the spread of AMR. In East Africa, antibiotic susceptibility data are sparse. To fill the gap, this study reports common microorganisms and their susceptibility patterns isolated from patients with UTI-like symptoms in Kenya, Tanzania and Uganda. Within each country, patients were recruited from three sites that were sociodemographically distinct and representative of different populations.

**Methods:**

UTI was defined by the presence of >10^4^ cfu/mL of one or two uropathogens in mid-stream urine samples. Identification of microorganisms was done using biochemical methods. Antimicrobial susceptibility testing was performed by the Kirby–Bauer disc diffusion assay. MDR bacteria were defined as isolates resistant to at least one agent in three or more classes of antimicrobial agents.

**Results:**

Microbiologically confirmed UTI was observed in 2653 (35.0%) of the 7583 patients studied. The predominant bacteria were *Escherichia coli* (37.0%), *Staphylococcus* spp. (26.3%), *Klebsiella* spp. (5.8%) and *Enterococcus* spp. (5.5%). *E. coli* contributed 982 of the isolates, with an MDR proportion of 52.2%. *Staphylococcus* spp. contributed 697 of the isolates, with an MDR rate of 60.3%. The overall proportion of MDR bacteria (*n* = 1153) was 50.9%.

**Conclusions:**

MDR bacteria are common causes of UTI in patients attending healthcare centres in East African countries, which emphasizes the need for investment in laboratory culture capacity and diagnostic algorithms to improve accuracy of diagnosis that will lead to appropriate antibiotic use to prevent and control AMR.

## Introduction

Increase of antimicrobial resistance (AMR) is currently considered one of the top 10 global public health threats.^1^ In 2019, there were an estimated 4.95 million deaths associated with antibacterial resistance (ABR) including 1.27 million deaths directly attributable to ABR.^2^ Among all world regions, sub-Saharan Africa has the largest burden of ABR-attributable deaths, although most contemporary ABR estimates in that region are based on incredibly sparse data.^2–4^ This serious threat requires a better assessment of ABR to understand the current and future burden of AMR and to direct the use of antibiotics (ABs) more effectively. This motivated the formation of the interdisciplinary consortium ‘Holistic Approach to Unravel Antibacterial Resistance in East Africa’ (HATUA), which aimed to explore the burden and drivers of ABR associated with urinary tract infections (UTIs) in three East African countries: Kenya, Tanzania and Uganda.^5^

UTI is an inflammatory response of the urothelium to bacterial invasion and is considered the most frequent community-acquired bacterial infection in the world, affecting more than 150 million people per year.^6,7^ In addition, UTIs are the third most frequent healthcare-associated infection (HAI), with approximately one-third of all deaths associated with HAIs.^8^ Globally, deaths attributable to and associated with ABR in UTIs in 2019 were approximately 65 000 and 250 000, respectively.^2,7^

UTI is the second most frequent reason for using ABs in the community, which can contribute to the emergence of MDR bacteria.^9^ The prevalence of MDR bacteria—defined as bacteria with non-susceptibility to at least one agent in ≥3 antimicrobial categories—associated with UTI has increased worldwide, thus limiting the therapeutic options for the treatment of infections caused by those microorganisms.^10–13^ A recent study in East Africa has estimated that the proportion of MDR uropathogens in 51%.^14^

In community-acquired UTIs, AB treatment is usually prescribed empirically. The selection of the empirical AB is based on surveillance mechanisms addressing the frequency of uropathogens and their antimicrobial resistance profiles. However, culture and susceptibility data for community UTI infections are unavailable in many low- and middle-income regions such as East Africa, mainly due to limited health service funding, and paucity of microbiology laboratory capacity including limited skilled personnel.^2–4^ These data are critical for prescribing the appropriate empirical AB, which could contribute to reducing the emergence of MDR bacteria and therefore UTI-associated complications, such as pyelonephritis or bacteraemia, through more effective treatment.^15^

The main goals of this study are, therefore, to describe the proportion of microbiologically confirmed UTIs in symptomatic patients who attended clinics in Kenya, Tanzania and Uganda, to characterize the main uropathogenic bacteria responsible and their AMR profiles, and to estimate the proportion of MDR bacteria associated with UTIs. The findings presented here can provide input for UTI empirical treatment guidelines in East Africa, helping to prevent the AMR-associated complications and deaths.

## Material and methods

### Study design, patient selection and sample size

The sample collection took place between April 2019 and November 2020 in Kenya, Tanzania and Uganda in different levels of health facilities and locations (Table S1, available as Supplementary data at *JAC-AMR* Online). In each country, three sites were selected for recruitment of patients that were representative of three sociodemographically distinct locations: (i) urban, economically advanced settings; (ii) remote villages in poorer areas; and (iii) pastoralist and neglected network areas.^5^ The sites were: Nairobi, Nanyuki and Makueni in Kenya; Mwanza, Mbeya and Kilimanjaro in Tanzania; and Mbarara, Nakapiripirit and Nakasongola in Uganda.

**Figure 1.**
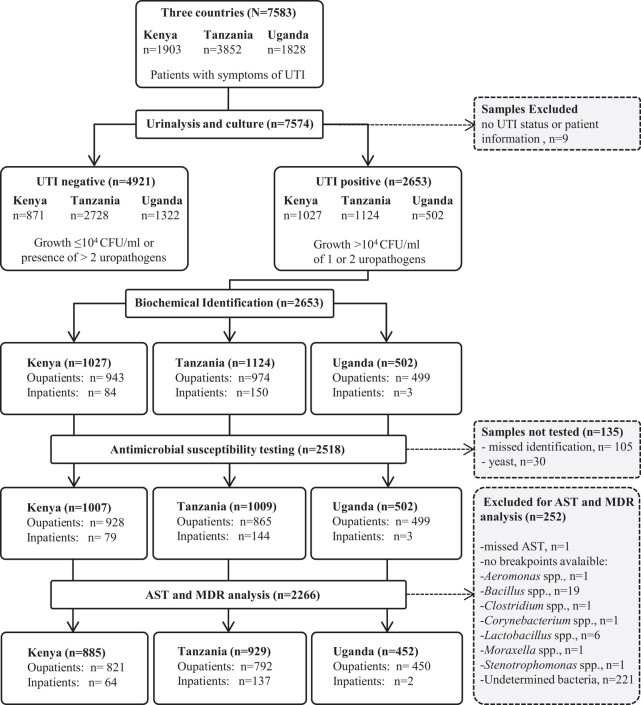
CONSORT diagram describing HATUA patient recruitment and processing and analysis of their urine samples.

The study included adults and children (≥2 years old) with signs and symptoms of UTI (detailed description for inclusion of patients is shown in Method S1). Self-collected mid-stream clean-catch urine samples were obtained from each patient, as described previously.^5^ Patients were classified according to their stay at the recruitment health facilities as outpatient (visits with no overnight stay) or inpatient (overnight or longer stay). A total of 7583 patients with symptomatic UTI were recruited from Kenya (*n* = 1903), Tanzania (*n* = 3852) and Uganda (*n* = 1828) (Figure 1).

### Urine culture and biochemical identification of isolates

A standard disposable sterile plastic loop was used to inoculate 1 μL or 10 μL of mid-stream urine sample onto cysteine/lactose/electrolyte-deficient (CLED) agar, sheep blood agar (SBA) and MacConkey agar plates (Oxoid, Basingstoke, UK).^16^ After 18–24 h of incubation at 37°C under aerobic conditions, cultures were quantified. Microbiologically confirmed UTI (hereafter UTI-positive sample) was defined by the presence of >10^4^ cfu/mL of one or two uropathogens. Contaminated samples (>10^4^ cfu/mL growth of more than two different uropathogens or any growth of <10^4^ cfu/mL) and those with no microbial growth were considered UTI negative. In samples containing two possible uropathogens, only the predominant or the most probable uropathogen (subject to evaluation by an experienced clinical microbiologist) was included in the analysis of the data.

In-house methods were used to identify Gram-negative bacteria and included: colonial morphology on CLED, SBA and MacConkey agar (Oxoid, Basingstoke, UK), and triple sugar iron agar, sulphur indole and motility, citrate, oxidase, urease, Voges–Proskauer and methyl red tests. Coagulase, catalase, bile aesculin and bacitracin/sulfamethoxazole disc susceptibility tests were used to confirm the presence of Gram-positive bacteria, which were identified using colonial morphology on SBA.

### Antimicrobial susceptibility testing (AST)

AST was performed by the conventional Kirby–Bauer disc diffusion method according to the CLSI M02 document.^17^ The discs (Oxoid, Basingstoke, UK) tested were ampicillin (10 µg), amoxicillin/clavulanic acid (20/10 µg), cefoxitin (30 µg), tetracycline (30 µg), trimethoprim (5 µg), ciprofloxacin (5 µg), gentamicin (10 µg), nitrofurantoin (100 µg), ceftriaxone (30 µg), ceftazidime (30 µg), erythromycin (15 µg), linezolid (10 µg) and vancomycin (30 µg). The susceptibility or non-susceptibility (resistance) to the tested ABs was determined by using the breakpoints (zone diameter interpretive criteria) indicated in the M100 document of CLSI guidelines, as further detailed in Method S2.^18^ Those isolates that showed intermediate resistance to a given AB were considered resistant to such AB. Prediction of possible ESBL producers was based on ceftazidime and/or ceftriaxone resistance, following the criteria indicated in the CLSI guidelines.^18^

### Definition and analysis of multidrug resistance

MDR bacteria were defined as isolates resistant to at least one agent in three or more classes of antimicrobial agents, following the ECDC guidelines, with some modifications as specified in Table S2 and Method S3.^10^ MDR rates were calculated by considering the number of MDR isolates divided by the total number of isolates.

### Patient characteristics

A questionnaire was conducted with all patients (or their parents/guardians), which captured sociodemographic factors including age, gender and other factors (e.g. education, marital status and household socioeconomic factors). Selected variables are shown in Table 1.

**Table 1.** Characteristics of the patients with symptoms of UTI at the time of recruitment

Variables/country	Kenya *n* (%)	Tanzania *n* (%)	Uganda *n* (%)	Total *n* (%)
Patient type				
* *Outpatient	1754 (92.4)	3552 (92.2)	1815 (99.5)	7121 (94.0)
* *Inpatient	144 (7.6)	300 (7.8)	9 (0.5)	453 (6.0)
Gender				
* *Male	348 (18.3)	1097 (28.5)	295 (16.2)	1740 (23.0)
* *Female	1550 (81.7)	2754 (71.5)	1529 (83.8)	5833 (77.0)
* *Missing	NA	1 (0.0)	NA	1 (0.0)
Age, years				
* *<18	79 (4.2)	343 (8.9)	62 (3.4)	484 (6.4)
* *18–24	493 (26.0)	718 (18.6)	573 (31.4)	1784 (23.6)
* *25–34	837 (44.1)	950 (24.7)	573 (31.4)	2360 (31.2)
* *35–44	294 (15.5)	550 (14.3)	304 (16.7)	1148 (15.2)
* *45–54	97 (5.1)	425 (11.0)	173 (9.5)	695 (9.2)
* *55–64	43 (2.3)	321 (8.3)	71 (3.9)	435 (5.7)
* *65–74	35 (1.8)	281 (7.3)	43 (2.4)	359 (4.7)
* *75 and above	20 (1.1)	261 (6.8)	22 (1.2)	303 (4.0)
* *Missing	0 (0)	3 (0.1)	3 (0.2)	6 (0.1)
Hospital level^a^				
* *Level 2 (low)	0 (0)	309 (8.0)	394 (21.6)	703 (9.3)
* *Level 3	384 (20.2)	2152 (55.9)	1023 (56.1)	3559 (47.0)
* *Level 4	486 (25.6)	366 (9.5)	143 (7.8)	995 (13.1)
* *Level 5/6 (high)	1028 (54.2)	1025 (26.6)	263 (14.4)	2316 (30.6)
* *Missing	0 (0)	0 (0)	1 (0.1)	1 (0.0)
Total	1898 (100.0)	3852 (100.0)	1824 (100.0)	7574 (100.0)

NA, not applicable.

^a^In all three countries, lower levels (1–3) refer to primary care, dispensaries or community health centres. Level 4 typically refers to primary referral facilities or specialist healthcare facilities. Level 5 (and 6 in Kenyan) are higher level/tertiary facilities.

### Data management and analysis

Data were captured using paper forms and electronically, using the Epicollect5 mobile application (https://five.epicollect.net).^19^ Urinalysis, AST and MDR data were linked to the questionnaire data using anonymous patient identifiers. AB susceptibility/MDR rates were calculated in R Statistical Software (v4.1.1; R Core Team 2021). Descriptive analysis and χ^2^ testing with false discovery rate correction were conducted in STATA 16 (StataCorp. 2019, Stata Statistical Software: Release 16. College Station, TX, USA).^20^

### Quality control

*Escherichia coli* ATCC 25922, *E. coli* NCTC 13353 (CTX-M-15 ESBL producer), *Staphylococcus aureus* ATCC 25923, *S. aureus* NCTC 13552 (*mecC*; MRSA), *Pseudomonas aeruginosa* ATCC 49189, *Proteus mirabilis* NCTC 10975 and *Enterococcus faecium* ATCC 51559 (*vanA*; vancomycin resistant) were used as reference strains for quality control of culture, biochemical identification and antimicrobial susceptibility tests.

## Ethics

The studies involving human participants were reviewed and approved by: University of St Andrews, UK (number MD14548, 10 September 2019); National Institute for Medical Research, Tanzania (number 2831, updated 26 July 2019); CUHAS/BMC Research Ethics and Review Committee (number CREC/266/2018, updated February 2019); Mbeya Medical Research and Ethics Committee (number SZEC-2439/R.A/V.1/303030); Kilimanjaro Christian Medical College, Tanzania (number 2293, updated 14 August 2019); Uganda National Council for Science and Technology (number HS2406, 18 June 2018); Makerere University, Uganda (number 514, 25 April 2018); and Kenya Medical Research Institute (04 June 2019, Scientific and Ethics Review Committee (SERU) number KEMRI/SERU/CMR/P00112/3865 V.1.2). The patients/participants provided their written informed consent to participate in this study.

## Results

### Study participants and samples

A CONSORT diagram of patient recruitment and analysis is shown in Figure 1. A total of 7583 urine samples from non-repetitive patients with suspected UTI were collected in Kenya, Tanzania and Uganda, of which 7574 were categorized as either UTI negative or UTI positive, according to the results of the urine cultures. Of a total of 2653 biochemically identified isolates, we obtained AST results for 2357 bacteria, which were subsequently included in the AST and MDR analysis.

### Demographic features

Participant characteristics are shown in Table 1. Most were adult outpatients (89.9%) and female (77%). The modal age category was 25 to 34 years, and those aged 18–34 years contributed more than half (54.7%) of the total sample.

### Proportion of microbiologically confirmed UTI

The overall proportion of microbiologically confirmed UTI across the three countries was 35.0%, being significantly higher in inpatients than in outpatients, in females, in patients recruited in higher-level facilities, and among patients over 65 years old (Table S3). Kenya reported a UTI proportion of 54.1%, which was higher than the proportions of 29.2% and 27.5% found in Tanzania and Uganda, respectively (Table S3).

### Identity of isolates from UTI

A total of 2653 isolates were characterized from urine samples of UTI-positive patients, 2416 from outpatients and 237 from inpatients, of which 94.9% corresponded to bacteria, 1.1% to yeast, and 4.0% to isolates whose biochemical identification was not available. Among the bacterial isolates (*n* = 2518), 62.7% and 37.3% were Gram-negative and Gram-positive bacteria, respectively, of which 91.2% (*n* = 2297) were identified to at least the genus level.

Considering the three countries together (Table 2), *E. coli* was the predominant species (37.0%), followed by *Staphylococcus* spp. (26.3%), *Klebsiella* spp. (5.8%) and *Enterococcus* spp. (5.5%). By country, Kenya showed a higher proportion of *Staphylococcus* spp. than Tanzania and Uganda, while Uganda showed a higher proportion of *E. coli* than Kenya and Tanzania. Globally, *E. coli*, *Staphylococcus* spp., *Enterococcus* spp. and *Pseudomonas* spp. were more represented in samples from outpatients than inpatients, while proportions of *Klebsiella* spp. and *Acinetobacter* spp. were higher in samples from inpatients (Table S4).

**Table 2.** Distribution of significant microorganisms isolated from specimens of symptomatic patients with UTI (UTI-positive patients), according to the country

Microbial isolates	Kenya			Tanzania			Uganda			All three countries		
	*n*	%^a^	Prev. (%)^b^	*n*	%	Prev. (%)	*n*	%	Prev. (%)	*n*	%	Prev. (%)
*E. coli*	317	30.9	16.7	402	35.8	10.4	263	52.4	14.4	982	37.0	13.0
*Klebsiella* spp.	0	0	0	90	8.0	2.3	64	12.7	3.5	154	5.8	2.0
*Proteus* spp.	69	6.7	3.6	13	1.2	0.3	15	3.0	0.8	97	3.7	1.3
*Acinetobacter* spp.	8	0.8	0.4	19	1.7	0.5	5	1.0	0.3	32	1.2	0.4
*Pseudomonas* spp.	8	0.8	0.4	41	3.6	1.1	1	0.2	0.1	50	1.9	0.7
Miscellaneous Gram-negative^c^	101	9.8	5.3	111	9.9	2.9	52	10.4	2.9	264	10.0	3.5
*Staphylococcus* spp.	387	37.7	20.4	220	19.6	5.7	91	18.1	5.0	698	26.3	9.2
*Enterococcus* spp.	86	8.4	4.5	58	5.2	1.5	3	0.6	0.2	147	5.5	1.9
Miscellaneous Gram-positive^d^	31	3.0	1.6	55	4.9	1.4	8	1.6	0.4	94	3.5	1.2
Yeast	2	0.2	0.1	28	1.1	0.7	0	0.0	0.0	30	1.1	0.4
Missing species data	18	1.8	0.9	87	7.7	2.3	0	0.0	0.0	105	4.0	1.4
Total	1027	100	54.1	1124	100	29.2	502	100	27.5	2653	100	35.0

^a^% = percentage of isolates corresponding to that species, from that country (for example, in the first column, calculated by 317/1027 × 100).

^b^Prev. = prevalence proportion (e.g. number of *E. coli* isolates with respect to the total number of urine specimens that were cultured in that country). For example, the third column is calculated by 317/1898 × 100.

^c^Across all three countries, this comprises *Aeromonas* spp. (*n* = 1), *Citrobacter* spp. (*n* = 16), *Enterobacter* spp. (*n* = 24), *Moraxella* spp. (*n* = 1), *Morganella* spp. (*n* = 6), *Pantoea* spp. (*n* = 2), *Providencia* spp. (*n* = 2), *Salmonella* spp. (*n* = 2), *Serratia* spp. (*n* = 4), *Shigella* spp. (*n* = 1), *Stenotrophomonas* spp. (*n* = 1) and undetermined Gram-negative bacteria (*n* = 113).

^d^Across all three countries, this comprises *Bacillus* spp. (*n* = 19), *Clostridium* spp. (*n* = 1), *Corynebacterium* spp. (*n* = 1), *Lactobacillus* spp. (*n* = 6), *Streptococcus* spp. (*n* = 50) and undetermined Gram-positive bacteria (*n* = 17).

### Regional burden of MDR in UTI pathogens

Of a total of 2266 isolates included in the AST and MDR analysis (Figure 1), 1153 (50.9%) were categorized as MDR. By country, MDR rates were similar in Tanzania (60.9%) and Uganda (57.5%), while Kenya had a lower MDR rate (36.9%) (Table 3). Considering all countries together, the proportion of uropathogens that were classified as MDR was significantly higher in isolates from inpatients, those recruited in lower-level facilities, and in male patients (Table 3). By country, MDR proportions in Kenya and Tanzania were higher in males than in females, but this relationship was reversed in Uganda. By pathogen, *Staphylococcus* spp. showed the higher rates of MDR (60.3%), followed by *E. coli* (52.2%), *Klebsiella* spp. (50.6%), *Enterococcus* spp. (38.1%) and other Enterobacterales (31.2%) (Table 4). Within each pathogen group, isolates from inpatients or males exhibited higher MDR rates than isolates from outpatients and females, respectively (Table 4).

**Table 3.** Prevalence of MDR bacteria in UTI-positive samples by country, and according to patient type, hospital level, gender and age

	MDR,^a^ *n* (%)			
Variable	Kenya *n* (%)	Tanzania *n* (%)	Uganda *n* (%)	All countries *n* (%)
Patient type				
* *Outpatient	291 (35.4)	466 (58.8)	258 (57.3)	1015 (49.2)
* *Inpatient	36 (56.3)	100 (73.0)	2 (100)	138 (68.0)
* *χ^2^, *P* value	9.7, *P* = 0.014	9.24, *P* = 0.019	0.25, *P* = 1.000	25.1, *P* < 0.001
Facility level				
* *Level 2/3	55 (35.7)	328 (59.2)	188 (54.3)	571 (54.2)
* *Level 4	109 (37.0)	55 (59.1)	26 (76.5)	190 (45.2)
* *Level 5/6	168 (38.1)	183 (64.9)	46 (63.9)	397 (49.9)
* *χ^2^, *P* value	0.27, *P* = 1.000	2.67, *P* = 0.728	7.62, *P* = 0.183	10.7, *P* < 0.001
Sex				
* *Male	40 (42.1)	174 (68.5)	30 (53.6)	244 (60.2)
* *Female	287 (36.3)	392 (58.2)	230 (58.1)	909 (48.9)
* *χ^2^, *P* value	1.41, *P* = 0.740	9.85, *P* = 0.030	0.24, *P* = 1.000	18.8, *P* < 0.001
Age, years				
* *<18	20 (45.5)	42 (64.6)	6 (66.7)	68 (57.3)
* *18–24	84 (34.1)	95 (56.9)	73 (52.1)	252 (45.5)
* *25–34	135 (35.3)	116 (62.0)	84 (57.5)	335 (46.8)
* *35–44	53 (39.3)	70 (54.3)	44 (66.7)	167 (50.6)
* *45–54	19 (50.0)	59 (59.0)	35 (60.3)	113 (57.6)
* *55–64	9 (56.3)	53 (66.3)	9 (64.3)	71 (64.5)
* *65–74	8 (44.4)	66 (66.0)	3 (30.0)	77 (60.2)
* *75 and above	4 (40.0)	65 (64.4)	4 (57.1)	73 (61.9)
* *χ^2^, *P* value	8.81, *P* = 0.740	6.71, *P* = 0.96	7.76, *P* = 1.000	35.3, *P* < 0.001
Total MDR	327 (36.9)	566 (60.9)	260 (57.5)	1153 (50.9)
Total isolates	885	929	452	2266

^a^MDR was defined as non-susceptibility to at least one antimicrobial agent in three or more antimicrobial categories, according to the ECDC guidelines with some modifications, as described in the Methods section (see Table S2).^10^ % is the prevalence of MDR, calculated by dividing the number of isolates that are MDR (*n*) by the number of isolates tested for MDR of each category and country.

**Table 4.** Prevalence of MDR bacteria in UTI-positive samples for selected species, according to patient type, age and gender

	MDR^a^ *n* (%)				
	*E. coli*	*Klebsiella* spp.	Other Enterobacterales^b^	*Staphylococcus* spp.	*Enterococcus* spp.
Patient type					
* *Outpatient	460 (50.8)	57 (44.9)	39 (28.3)	389 (59.5)	45 (34.4)
* *Inpatient	53 (69.7)	21 (77.8)	9 (56.3)	31 (72.1)	11 (68.8)
* *χ^2^, *P* value	9.36, *P* = 0.006	8.36, *P* = 0.020	4.01, *P* = 0.120	2.11, *P* = 0.803	5.58, *P* = 0.064
Age					
* *Adult	481 (51.7)	71 (49.7)	43 (30.9)	403 (59.9)	52 (37.7)
* *Child	31 (62.0)	6 (60.0)	5 (33.3)	17 (70.8)	4 (44.4)
* *χ^2^, *P* value	1.64, *P* = 0.367	0.09, *P* = 1.000	0.01, *P* = 1.000	0.71, *P* = 0.970	0.01, *P* = 1.000
Gender					
* *Male	107 (71.3)	37 (63.8)	23 (44.2)	43 (64.2)	17 (58.6)
* *Female	406 (48.8)	41 (42.7)	25 (24.5)	377 (59.8)	39 (33.1)
* *χ^2^, *P* value	25.7, *P* < 0.001	5.61, *P* = 0.048	5.35, *P* = 0.113	0.39, *P* = 0.970	5.14, *P* = 0.064
Total MDR	513 (52.2)	78 (50.6)	48 (31.2)	420 (60.3)	56 (38.1)
Total isolates	982	154	154	697	147

^a^MDR was defined as non-susceptibility to at least one antimicrobial agent in three or more antimicrobial categories, according to the ECDC guidelines with some modifications, as described in the Methods section (see Table S2).^10^ % is the prevalence of MDR, calculated by dividing the number of isolates that are MDR (*n*) by the number of isolates tested for MDR of each category and selected species.

^b^Other Enterobacterales includes *Citrobacter*, *Enterobacter*, *Morganella*, *Proteus*, *Providencia*, *Pantoea*, *Salmonella*, *Serratia* and *Shigella* species.

### AB susceptibility and MDR in Enterobacterales

The overall resistance rates of Enterobacterales ranged from 71.6% for trimethoprim to 7.5% for nitrofurantoin. The proportion of isolates with an ESBL and MDR were 31.4% and 49.5%, respectively (Table 5). Within bacterial groups, the resistance rates of the *E. coli* isolates ranged from 74.4% for trimethoprim to 4.1% for nitrofurantoin (Table 5), with an ESBL and MDR proportion of 29.3% and 52.2%, respectively. *Klebsiella* spp. isolates exhibited resistance rates between 93.5% for ampicillin to 14.3% for nitrofurantoin (Table 5) and ESBL and MDR rates of 53.9% and 50.6%, respectively. The resistance rates of other Enterobacterales ranged from 61.8% for trimethoprim to 15.1% for gentamicin, displaying ESBL and MDR rates of 21.7% and 30.9%, respectively.

**Table 5.** AB non-susceptibility, ESBL and MDR rates of Enterobacterales and relevant Gram-positive uropathogens

	Enterobacterales		*E.coli*		*Klebsiella* spp.		Other Enterobacterales^a^		*Staphylococcus* spp.		*Enterococcus* spp.	
	%^b^	*n* ^c^	%	*n*	%	*n*	%	*n*	%	*n*	%	*n*
Ampicillin	65.9	1289	64.5	981	93.5	154	46.8	154	NA^d^	NA	NA	NA
Amoxicillin/clavulanic acid	40.7	1276	38.1	970	60.1	153	37.3	153	NA	NA	NA	NA
Ceftazidime	24.0	1277	22.7	971	41.2	153	15.7	153	NA	NA	NA	NA
Ceftriaxone	30.2	1287	28.5	979	51.3	154	20.1	154	NA	NA	NA	NA
Ciprofloxacin	44.8	1289	45.8	982	43.5	154	39.2	153	38.2	696	40.1	147
Gentamicin	21.5	1170	22.1	900	24.8	129	14.9	141	20.9	669	NA	NA
Nitrofurantoin	7.5	1286	4.1	978	14.3	154	22.7	154	5.5	692	9.5	147
Trimethoprim	71.6	1289	74.4	981	63.0	154	62.3	154	81.8	696	NA	NA
Cefoxitin (methicillin resistance)	NA	NA	NA	NA	NA	NA	NA	NA	39.7	692	NA	NA
Linezolid	NA	NA	NA	NA	NA	NA	NA	NA	15.9	661	8.8	136
Erythromycin	NA	NA	NA	NA	NA	NA	NA	NA	72.5	662	69.8	139
Tetracycline	NA	NA	NA	NA	NA	NA	NA	NA	48.0	671	50.3	145
Vancomycin	NA	NA	NA	NA	NA	NA	NA	NA	NA	NA	37.2	137
ESBL^e^	31.4	1290	29.3	982	53.9	154	22.1	154	NA	NA	NA	NA
MDR^f^	49.5	1290	52.2	982	50.6	154	31.2	154	60.3	697	38.1	147

^a^Other Enterobacterales includes *Citrobacter*, *Enterobacter*, *Morganella*, *Proteus*, *Providencia*, *Pantoea*, *Salmonella*, *Serratia* and *Shigella* species.

^b^% is the frequency of non-susceptible isolates, expressed as percentage.

^c^ *n* is the total number of isolates that were tested for a specific AB.

^d^Not applicable (NA), the AB was not tested in those isolates.

^e^Possible producers of ESBL were determined by considering the resistance to the ABs ceftazidime and ceftriaxone, according to CLSI guidelines.^18^

^f^MDR was defined as non-susceptibility to at least one antimicrobial agent in three or more antimicrobial categories, according to the ECDC guidelines with some modifications, as described in the Methods section.^10^

*E. coli* from Kenya were less likely to be resistant to ampicillin, amoxicillin/clavulanic acid, trimethoprim, ciprofloxacin, ceftriaxone and ceftazidime than those from Tanzania and Uganda, while in Tanzania, *E. coli* resistance to nitrofurantoin was higher than the other countries (Table S5). In addition, MDR and ESBL were less common among *E. coli* isolates from Kenya than those from Tanzania and Uganda, while MDR *Klebsiella* spp. were less represented in Uganda than in Tanzania. Regarding other Enterobacterales, isolates from Kenya were significantly less likely to be resistant to ampicillin, amoxicillin/clavulanic acid, ceftriaxone and ceftazidime than those from Tanzania and Uganda, and also showed lower ESBL and MDR rates. Ugandan isolates showed significantly higher rates of resistance to nitrofurantoin than isolates from other countries (Table S5).

The proportion of resistant isolates was generally higher in inpatients (Table S6) than outpatients (Table S7). Prevalence of ESBL and MDR among inpatient isolates was higher among *E. coli*, *Klebsiella* spp. and other Enterobacterales than those from outpatients (Table S6 and S7).

### AB susceptibility and MDR in staphylococci and enterococci

The proportion of resistant *Staphylococcus* spp. isolates ranged from 5.5% for nitrofurantoin to 81.8% for trimethoprim, with an MDR prevalence of 60.3% (Table 5). Cefoxitin resistance, indicating methicillin resistance, among staphylococci was 37.5%, 42.4% and 42.9% for Kenya, Tanzania and Uganda respectively (Table S8). *Staphylococcus* spp. from Kenya showed a higher proportion of linezolid-resistant isolates (23.4%) than the other two countries (5.5%–7.5%) (Table S8). Isolates from Tanzania had the greatest proportion with MDR (72.3%).

For *Enterococcus* spp., the overall resistance rates ranged from 8.8% for linezolid to 69.8% for erythromycin, with an MDR prevalence of 38.1% (Table 5). Comparisons among countries revealed that *Enterococcus* sp. isolates from Kenya were less resistant to tetracycline and nitrofurantoin, and more resistant to linezolid than isolates from Tanzania and Uganda, with no significant differences in MDR rates (Table S8).

*Staphylococcus* spp. isolates from inpatients (Table S9) showed higher resistance than isolates from outpatients (Table S10), except for the ABs ciprofloxacin, trimethoprim and tetracycline, also displaying increased MDR (72.1% versus 59.5%) (Tables S9 and S10). *Enterococcus* spp. from inpatients were more resistant to ciprofloxacin, erythromycin and tetracycline, and showed higher MDR than outpatients (68.8% versus 34.4%) (Tables S9 and S10).

## Discussion

This study samples the patterns of ABR in bacteria associated with UTIs in symptomatic patients in East Africa. Our main finding is that rates of ABR of the main uropathogens isolated from UTIs (*E. coli*, *Staphylococcus* spp., *Klebsiella* spp. and *Enterococcus* spp.) are severely high. Further, approximately half of the bacterial pathogens isolated from UTIs have MDR. That rate was much higher among inpatients (which we assume are predominantly hospital-acquired UTI) than in outpatients (which we assume are predominantly community-acquired UTI), as has been described previously.^21,22^ These alarming data provide further empirical evidence to enrich the findings of recent studies describing the high morbidity and mortality burden from ABR in Eastern sub-Saharan Africa.^2^

The high proportion of MDR in UTI could suggest a previous record of inappropriate AB use in Kenya, Tanzania and Uganda, which is often considered to be one of the key drivers of AMR. This could be caused by: (i) the scarcity of microbiology and AB susceptibility data in this region, which can hamper the management of more appropriate empirical treatment for UTIs; and (ii) AB self-treatment and the prevalence of over-the-counter sales of ABs in the community, widespread in low-and middle-income countries (LMICs).^2–4,23–25^ Suboptimal management of treatment and the community transmission of MDR bacteria promoted by crowded and less sanitary living conditions, more common in LMICs, could explain the high proportions of MDR bacteria and the tendency in the study cohort to come straight to clinic.^26,27^

In addition, we found differences among countries, with Kenya presenting a lower percentage of MDR bacteria (36.9%) than Tanzania (60.9%) and Uganda (57.5%). Worthy of special attention are the high MDR rates of *E. coli* (>66.0%) found in Tanzania and Uganda, as well as MDR *Klebsiella* (62.2%), *Staphylococcus* (72.3%) and *Enterococcus* (46.6%) species observed in Tanzania, which were much higher than in the other countries. These results emphasize the importance of implementing or reviewing country-specific empirical AB recommendations, which could increase AB efficacy and reduce the burden of AMR according to the resistance rates of each country.^28^

Globally, our results fill a crucial data gap, which we hope will: (i) feed into guidelines for UTI empirical treatment; (ii) provide vital surveillance data for East Africa and indeed the wider sub-Saharan region, a region with one of the highest ABR-mortality burdens in the world; and (iii) contribute to development of interventions to monitor and counter the threat of ABR across the region through improved diagnostics and surveillance.

The sparsity of data about the prevalence of resistance for key AB–pathogen combinations in LMICs is a limiting factor for drafting empirical treatment guidelines, which can promote appropriate prescription hence hindering the selection of the resistant pathogens.^2–4^ In this study, we have found a high prevalence of the most insidious AB–pathogen combinations, i.e. third-generation cephalosporin (3GC)-resistant *E. coli* (29.3%), fluoroquinolone-resistant *E. coli* (45.8%), 3GC-resistant *Klebsiella* spp. (53.9%), methicillin-resistant staphylococci (39.7%), fluoroquinolone-resistant *Enterococcus* spp. (40.1%) and VRE (37.2%). However, we observed systematic variations across country settings, with the Kenyan samples showing the lowest rate of resistance to these ABs, which suggest that recommendations for using a specific empirical AB should be tailored according to each country.^28^ The high proportion of fluoroquinolone-resistant *E. coli* and fluoroquinolone-resistant *Enterococcus* spp. found in this study, which are in the top six of the most lethal AB–pathogen combinations in UTI, advise against the empirical use of this AB, whose use in treatment of uncomplicated UTI is no longer recommended by WHO.^7,29,30^ The clinical guidelines of Tanzania and Uganda recommended ciprofloxacin as first- or second-line ABs for the treatment of uncomplicated UTI in outpatients, which could explain the higher fluoroquinolone resistance observed in these two countries than those observed in Kenya.^31–33^

MRSA was the most lethal drug–pathogen combination in 2019 in the world, being in the top 10 of resistance-attributable deaths in UTI.^2,7^ Although in our study staphylococci were not analysed to species level, we found an overall rate of methicillin (cefoxitin) resistance of 39.7%. This contrasts with global estimations in sub-Saharan Africa, which have been recently described as one of the lowest in the world (5%).^2^ Our study has revealed *Staphylococcus* spp. as the second most frequent genus in UTI, which is in line with current evidence that points towards a major role of this species as a common cause of UTI.^34–36^ Although we cannot rule out contamination with *Staphylococcus* spp. in UTI samples, the fact that nearly two of every three isolates were MDR, and ∼40% were resistant to cefoxitin, should be considered for managing *Staphylococcus* spp. as true causative agents of UTI.

Amoxicillin/clavulanic acid is among the ABs commonly used to treat uncomplicated UTI in East Africa. In this study, we found a high level of resistance (37.3%–47.1%) to amoxicillin/clavulanic acid in Enterobacterales, which could endanger its future empirical use for treatment of UTIs, as happened with amoxicillin alone, the use of which in uncomplicated UTI is no longer recommended.^30,37^

In addition, the overall resistance to the folate pathway inhibitor trimethoprim was exceptionally high (53.9%–74.4%) in isolates from order Enterobacterales, while resistance to nitrofurantoin was low. This trend has been reported in UTIs worldwide, which has led to the prioritization of the use of nitrofurantoin over trimethoprim as the first-line treatment for UTI, including in East Africa.^31–33,38^ In 2021, however, the WHO added single-agent trimethoprim as a recommendation for the treatment of uncomplicated UTI, whose empirical use in East Africa (with a trimethoprim resistance rate in *E. coli* of up to 84.1% in Tanzania), would make that AB poorly effective for the treatment of UTI in that region.^30^

The study has some limitations. In the design of the HATUA we endeavoured to provide a consistent study framework across the three countries and the three sites within each country where patients were recruited and their samples were processed and analysed. Standardization of methods and operating procedures were applied across the consortium and used by the Kenyan, Tanzanian and Ugandan chapters of HATUA.^5^ However, even with these in place we cannot rule out that some biases in sampling practices or patient populations studied will have occurred.

Within each country, three sites were chosen that had three distinct sociodemographic characteristics and represented a different type of site. This was done in order to capture the burden of AMR in UTIs across different community settings in each country. Whilst each country selected sites that were representative of each site type, and provided some level of sociodemographic comparability across countries for the study, there is variation that a study of this scale introduces that means that the populations are not equivalent due to geographic, climatic, ethnic and cultural factors. In this regard we note that across the three countries there are differences in the demographic profiles of the patients recruited. For example, in Kenya more recruitment occurred at higher-level health facilities, and the cohort had a greater proportion of patients under the age of 35 years in comparison with those of the other countries. We cannot therefore exclude the introduction of bias that may influence some of the observed microbiological results and some of the differences seen between countries. Recognizing this, the interpretation of the results should reflect that they do not necessarily represent true country-level differences across the region, as the sampling within the countries is limited to three sites and is not representative of the countries as a whole.

With such a large, multi-site study, and need for comparability, there have been some inevitable trade-offs between depth and breadth, and as a result for most of the isolates, only their identification to genus level is shown. As samples from outpatients were self-collected, there was a risk of contamination in the samples, which could help explain the high levels of *Staphylococcus* spp. found in this study. Although a wide range of the most commonly used/relevant ABs for UTI in the region was tested, this did not include all possible ABs, which could have led to an underestimation of the true MDR proportions, and therefore our estimates of the burden of AMR on patients with UTIs are conservative.

### Conclusions

This multi-site standardized study describes how approximately half of UTI patients that attended our recruitment centres in Kenya, Tanzania and Uganda exhibit MDR bacteria. Several of the most hazardous AB–pathogen combinations (3GC- and fluoroquinolone-resistant *E. coli*; methicillin-resistant staphylococci; 3GC-resistant *Klebsiella pneumoniae*; VRE; MDR bacteria) were detected at high proportions in UTI, which severely limits the effectiveness of currently used ABs to treat this common infection. These findings should feed directly into guidelines for empirical AB treatment of UTI in East Africa. More broadly, we emphasize the need for urgent investment in routine AMR surveillance programmes, expansion of diagnostic laboratory capacities and diagnostic algorithms to facilitate antimicrobial stewardship and call for greater commitment from policymakers to counter the threat of AMR.

## Supplementary Material

dlae019_Supplementary_Data

## Members of the HATUA consortium

Everyone listed on the title page, plus: Blandina T. Mmbaga (Kilimanjaro Clinical Research Institute and Kilimanjaro Christian Medical University College, Tanzania), John Mwaniki (KEMRI, Kenya), Stella Neema (Makerere University, Uganda), Joseph R. Mwanga (Catholic University of Health and Allied Sciences, Tanzania), Arun Gonzales Decano (University of St Andrews, UK), V. Anne Smith (University of St Andrews, UK), Alison Elliott (London School of Hygiene & Tropical Medicine, UK), Gibson Kibiki (Africa Research Excellence Fund) and David Aanensen (University of Oxford, UK).

## Supplementary data

Tables S1 to S9 are available as Supplementary data at *JAC-AMR* Online.
