# Supplementary material for: Predominance of multidrug-resistant bacteria causing urinary tract infections among symptomatic patients in East Africa: a call for action
Source: JAC Antimicrob Resist. 2024 Feb 14;6(1):dlae019. doi: 10.1093/jacamr/dlae019 (PMC10873138; doi:10.1093/jacamr/dlae019)
Supplement: dlae019_Supplementary_Data [file dlae019_supplementary_data.docx]

**Appendix. Supplementary materials**

**Method S1. Patient selection/Case definition**

The study included adults and children (≥ 2 years) with signs and symptoms of urinary tract infections. Patients attended to the health facilities to seek treatment for UTI-like symptoms or for other causes that gave the doctor reason to believe that they might also (or actually) have a UTI.

Specifically, the study enrolled: (a) pregnant women with fever and at least one of the following symptoms, lower abdominal pain, flank/back pain, or strong-smelling urine; (b) pregnant women with at least one urinary symptom, i.e., dysuria, pyuria or haematuria; (c) non-pregnant women or men with fever and at least one of the following symptoms, lower abdominal pain, flank/back pain or strong-smelling urine; (d) non-pregnant women or men with at least one urinary symptom, i.e., frequency, dysuria, pyuria, haematuria or urgency.^1-3^ In addition, the study enrolled all children aged two years and above that complied with at least one of the following criteria: (a) fever and at least one of: vomiting, abdominal pain, flank/back pain, strong-smelling urine or enuresis; (b) at least one of these urinary symptoms: dysuria, pyuria, hematuria, urgency or frequency; (c) or child with at least two of: costovertebral angle tenderness, abdominal or suprapubic tenderness to palpation, palpable bladder or dribbling/poor stream.^4^

**Method S2. Antimicrobial susceptibility testing (AST)**

The susceptibility or non-susceptibility (resistance) to the tested antibiotics was determined by using the breakpoints (zone diameter interpretive criteria) indicated in the CLSI guidelines with the following modifications.^5^ For nitrofurantoin and linezolid, the European Committee on Antimicrobial Susceptibility Testing breakpoints were used.^6^ For *Acinetobacter* spp., susceptibility to amoxicillin/clavulanic acid and trimethoprim was determined using the breakpoints for *Enterobacterales*. For *Staphylococcus* spp., methicillin resistance (non-susceptibility to cefoxitin) was calculated using the breakpoints for *S. epidermidis* and *Staphylococcus* spp. For *Pseudomonas* spp., breakpoints for *Pseudomonas aeruginosa* were applied.

**Method S3. Definition and analysis of Multidrug resistance (MDR)**

MDR bacteria were defined as isolates resistant to at least one agent in three or more classes of antimicrobial agents, following the ECDC guidelines, with some modifications.^7^ Thus, nitrofurantoin and trimethoprim, two antibiotics routinely used for treating UTIs that are not included in the ECDC tables, were also considered for estimating MDR (Table S2).^7^ In addition, for those species/genera not incorporated in the ECDC, i.e. *Salmonella*, *Shigella* and *Streptococcus*, the MDR rates were calculated following the same rule as described above, but considering the resistance to a selected pool of tested antibiotics (Table S2).

Those *Staphylococcus* spp. isolates that were resistant to cefoxitin (methicillin-resistant), were not automatically considered MDR, and were subjected to the rule of being resistant to at least one agent in three or more classes of antimicrobial agents. In those isolates with intrinsic resistance to a given antibiotic, such antibiotic was not considered for calculating MDR. By means of this approach, isolates from UTIs were classified as MDR or non-MDR. MDR rates were calculated by considering the number of MDR isolates divided by the total isolates where MDR data were obtained.

**Table S1.** Patient recruitment sites and healthcare facilities in Kenya, Tanzania, and Uganda.

| **Country/site** | **Number of facilities** | **Source of funding** | **Levels recruited from^a^** |
| --- | --- | --- | --- |
| **Kenya** |  |  |  |
| Makueni | 1 | Public | 5 |
| Nairobi | 4 | Public and private | 3, 4, 5, 6 |
| Nanyuki | 1 | Public | 4 |
| **Tanzania** |  |  |  |
| Kilimanjaro/Moshi | 3 | Public and private | 2, 3, 5 |
| Mbeya | 2 | Public and private | 3, 4 |
| Mwanza | 5 | Public and private | 2, 3, 5 |
| **Uganda** |  |  |  |
| Mbarara | 3 | Public | 3, 5 |
| Nakapiripirit | 3 | Public | 2, 3 |
| Nakasongola | 3 | Public and private | 3, 4 |

^a^ Levels of facilities are identified in each country following the Kenya Health Policy 2014-2013 (<http://publications.universalhealth2030.org/uploads/kenya_health_policy_2014_to_2030.pdf>), Tanzania fifth health sector strategic plan (HSSP V) 2021-2026 (<https://p4h.world/en/node/11813#:~:text=2026%20%7C%20P4H%20Network-,Tanzania%20fifth%20health%20sector%20strategic%20plan%20(HSSPV)%2D%202021%2D2026,coverage%20(UHC)%20by%202030)> and the Ugandan Hospital and Health Centre IV census survey 2014

In all three countries, lower levels (1-3) refer to primary care, dispensaries or community health centres. Level 4 typically refers to primary referral facilities or specialist health care facilities. Level 5 (and 6 in Kenyan) are higher level/tertiary facilities.

**Table S2.** Antibiotics considered for MDR calculations.

| **Gram negative** | **Amoxicillin/**  **clavulanate**  **(AMC)** | **Ampicillin**  **(AMP)** | **Ceftazidime/**  **Ceftriaxone**  **(CAZ/CRO)** | **Ciprofloxacin**  **(CIP)** | **Gentamicin**  **(GEN)** | **Nitrofurantoin**  **(NIT)** | **Trimethoprim**  **(TMP)** |  | |
| --- | --- | --- | --- | --- | --- | --- | --- | --- | --- |
| ***E. coli*** | AMC | AMP | CAZ/CRO | CIP | GEN | NIT | TMP |  | |
| ***Shigella* spp.** | AMC | AMP | CAZ/CRO | CIP | GEN | NIT | TMP |  | |
| ***Proteus* spp.** | AMC | AMP | CAZ/CRO | CIP | GEN | NIT | TMP |  | |
| ***Salmonella* spp.** | AMC | AMP | CAZ/CRO | CIP | GEN | NIT | TMP |  | |
| ***Serratia* spp.** | - | - | CAZ/CRO | CIP | GEN | NIT | TMP |  | |
| ***Klebsiella* spp.** | AMC | - | CAZ/CRO | CIP | GEN | NIT | TMP |  | |
| ***Citrobacter* spp.** | **-** | - | CAZ/CRO | CIP | GEN | NIT | TMP |  | |
| ***Enterobacter* spp.** | - | - | CAZ/CRO | CIP | GEN | NIT | TMP |  | |
| ***Morganella* spp.** | **-** | **-** | CAZ/CRO | CIP | GEN | NIT | TMP |  | |
| ***Pantoea* spp.** | - | - | CAZ/CRO | CIP | GEN | NIT | TMP |  | |
| ***Providencia* spp.** | - | - | CAZ/CRO | CIP | GEN | NIT | TMP |  | |
| ***Acinetobacter* spp.** | - | - | CAZ/CRO | CIP | GEN | - | TMP |  | |
| ***Pseudomonas* spp.** | - | - | CAZ | CIP | GEN | - | - |  | |
|  |  |  |  |  |  |  |  |  |  |
|  |  |  |  |  |  |  |  |  |  |
| **Gram positive** | **Cefoxitin**  **(FOX)** | **Erythromycin**  **(ERY)** | **Linezolid**  **(LNZ)** | **Ciprofloxacin**  **(CIP)** | **Gentamicin**  **(GEN)** | **Nitrofurantoin**  **(NIT)** | **Trimethoprim**  **(TMP)** | **Tetracycline**  **(TCY)** | **Vancomycin**  **(VAN)** |
| ***Staphylococcus* spp.** | FOX | ERY | - | CIP | GEN | NIT | TMP | TCY | - |
| ***Enterococcus* spp.** | - | ERY | LNZ | CIP | - | NIT | - | TCY | VAN |
| ***Streptococcus* spp.** | **-** | ERY | LNZ | - | - | NIT | - | TCY | VAN |

**Table S3.** Prevalence of UTI according to patient type, gender, hospital level age, and country.

|  | **N/% with UTI** | | | |
| --- | --- | --- | --- | --- |
| **Variables/Country** | **Kenya**  **n (%)^a^** | **Tanzania**  **n (%)^a^** | **Uganda**  **n (%)^a^** | **All 3 countries**  **n (%)^a^** |
| **Patient type** |  |  |  |  |
| Outpatient | 943(53.8) | 974(27.4) | 499(27.5) | 2416(33.9) |
| Inpatient | 84(58.3) | 150(50.0) | 3(33.3) | 237(52.3) |
| χ^2 ,^ p value^2^ | 0.94, p=0.690 | 67.2, p<0.001 | 0.1, p=1.000 | 62.48, p<0.001 |
| **Gender** |  |  |  |  |
| Male | 111(31.9) | 305(27.8) | 64(21.7) | 480(27.6) |
| Female | 916(59.1) | 818(29.7) | 438(28.6) | 2172(37.2) |
| χ2, p value^2^ | 69.1, p<0.001 | 15.2, p=0.004 | 7.7, p=0.217 | 190.6, p<0.001 |
| **Hospital level** |  |  |  |  |
| Level 2 | 0 | 88(28.5) | 126(32.0) | 214(30.4) |
| Level 3 | 162(42.2) | 585(27.2) | 259(25.3) | 1006(28.3) |
| Level 4 | 336(69.1) | 104(28.4) | 37(25.9) | 477(47.9) |
| Level 5/6 | 529(51.4) | 347(33.9) | 80(30.4) | 956(41.3) |
| χ2, p value^2^ | 83.6, p<0.001 | 3.8, p=0.312 | 5.6, p=0.14 | 56.7, p<0.001 |
| **Age** |  |  |  |  |
| <18 | 51(64.6) | 75(21.9) | 11(17.7) | 137(28.3) |
| 18-24 | 286(58.0) | 211(29.4) | 155(27.1) | 652(36.5) |
| 25-34 | 436(52.1) | 243(25.6) | 159(27.8) | 838(35.5) |
| 35-44 | 148(50.3) | 150(27.3) | 75(24.7) | 373(32.5) |
| 45-54 | 49(50.5) | 111(26.1) | 62(35.8) | 222(31.9) |
| 55-64 | 19(44.2) | 95(29.6) | 17(23.9) | 131(30.1) |
| 65-74 | 23(65.7) | 111(39.5) | 12(27.9) | 146(40.7) |
| 75 and above | 15(75.0) | 127(48.7) | 9(40.9) | 151(49.8) |
| χ^2^ , p value^b^ | 17.2, p=0.045 | 80.2, p<0.001 | 12.7, p=0.22 | 56.6, p<0.001 |
| **Total** | 1027(54.1) | 1124(29.2) | 502(27.5) | 2653(35.0) |

**^a^** % is the prevalence of UTI positive samples, calculated by dividing the number of UTI positive samples by the total number of urine samples cultured (n). A sample was considered UTI-positive if presented >10^4^ cfu/mL of one or two uropathogens.

^b^ Chi-squared testing adjusted for false discovery rate (as described in the methods).

**Table S4**. Distribution of significant microorganisms isolated from specimens of symptomatic patients with UTI (UTI positive patients), according to the country and the type of patient (Outpatient or Inpatient).

| **Country** | **Kenya**  **Outpatient** | | | **Kenya**  **Inpatient** | | | **Tanzania Outpatient** | | | **Tanzania**  **Inpatient** | | | **Uganda**  **Outpatient** | | | **Uganda**  **Inpatient** | | | **All 3 countries Outpatient** | | | **All 3 countries Inpatient** | | |
| --- | --- | --- | --- | --- | --- | --- | --- | --- | --- | --- | --- | --- | --- | --- | --- | --- | --- | --- | --- | --- | --- | --- | --- | --- |
| **Microbial Isolates** | n | %^a^ | Prev  (%)^b^ | n | % | Prev  (%) | n | % | Prev  (%) | n | % | Prev  (%) | n | % | Prev  (%) | n | % | Prev  (%) | n | % | Prev  (%) | n | % | Prev  (%) |
| *Escherichia coli* | 294 | 31.2 | 16.9 | 23 | 27.4 | 16.0 | 350 | 35.9 | 9.9 | 52 | 34.7 | 17.3 | 262 | 52.5 | 14.4 | 1 | 33.3 | 11.1 | 906 | 37.5 | 12.8 | 76 | 32.1 | 16.8 |
| *Klebsiella* spp. | 0.0 | 0.0 | 0.0 | 0.0 | 0.0 | 0.0 | 64 | 6.6 | 1.8 | 26 | 17.3 | 8.7 | 63 | 12.6 | 3.5 | 1 | 33.3 | 11.1 | 127 | 5.3 | 1.8 | 27 | 11.4 | 6.0 |
| *Proteus* spp. | 66 | 7.0 | 3.8 | 3 | 3.6 | 2.1 | 12 | 1.2 | 0.3 | 1 | 0.7 | 0.3 | 15 | 3.0 | 0.8 | 0 | 0.0 | 0.0 | 93 | 3.8 | 1.3 | 4 | 1.7 | 0.9 |
| *Acinetobacter* spp. | 7 | 0.7 | 0.4 | 1 | 1.2 | 0.7 | 14 | 1.4 | 0.4 | 5 | 3.3 | 1.7 | 5 | 1.0 | 0.3 | 0 | 0.0 | 0.0 | 26 | 1.1 | 0.4 | 6 | 2.5 | 1.3 |
| *Pseudomonas aeruginosa* | 6 | 0.6 | 0.3 | 2 | 2.4 | 1.4 | 25 | 2.6 | 0.7 | 16 | 10.7 | 5.3 | 1 | 0.2 | 0.1 | 0 | 0.0 | 0.0 | 32 | 1.3 | 0.4 | 18 | 7.6 | 4.0 |
| Miscellaneous  Gram-negative^c^ | 87 | 9.2 | 5.0 | 14 | 16.7 | 9.7 | 94 | 9.7 | 2.6 | 17 | 11.3 | 5.7 | 51 | 10.2 | 2.8 | 1 | 33.3 | 11.1 | 232 | 9.6 | 3.3 | 32 | 13.5 | 7.1 |
| *Staphylococcus* spp. | 359 | 38.1 | 20.6 | 28 | 33.3 | 19.4 | 205 | 21.0 | 5.8 | 15 | 10.0 | 5.0 | 91 | 18.2 | 5.0 | 0 | 0.0 | 0.0 | 655 | 27.1 | 9.2 | 43 | 18.1 | 9.5 |
| *Enterococcus* spp. | 81 | 8.6 | 4.7 | 5 | 6.0 | 3.5 | 47 | 4.8 | 1.3 | 11 | 7.3 | 3.7 | 3 | 0.6 | 0.2 | 0 | 0.0 | 0.0 | 131 | 5.4 | 1.9 | 16 | 6.8 | 3.5 |
| Miscellaneous  Gram-positive^d^ | 28 | 3.0 | 1.6 | 3 | 3.6 | 2.1 | 54 | 5.5 | 1.5 | 1 | 0.7 | 0.3 | 8 | 1.6 | 0.4 | 0 | 0.0 | 0.0 | 90 | 3.7 | 1.3 | 4 | 1.7 | 0.9 |
| Yeast | 2 | 0.2 | 0.1 | 0 | 0.0 | 0.0 | 25 | 2.6 | 0.7 | 3 | 2.0 | 1.0 | 0 | 0.0 | 0.0 | 0 | 0.0 | 0.0 | 27 | 1.1 | 0.4 | 3 | 1.3 | 0.7 |
| Missing species data | 13 | 1.4 | 0.7 | 5 | 6.0 | 3.5 | 84 | 8.6 | 2.4 | 3 | 2.0 | 1.0 | 0 | 0.0 | 0.0 | 0 | 0.0 | 0.0 | 97 | 4.0 | 1.4 | 8 | 3.4 | 1.8 |
| Total | 943 | 100 | 53.8 | 84 | 100 | 58.3 | 974 | 100 | 27.4 | 150 | 100 | 50.0 | 499 | 100 | 27.5 | 3 | 100 | 33.3 | 2416 | 100 | 33.9 | 237 | 100 | 52.3 |

^a^%= percentage of isolates corresponding to that species, from that country and patient type (for example in the first column, calculated by 294/943*100)

^b^Prev= prevalence proportion (e.g. number of *E. coli* isolates with respect to the total number of urine specimens that were cultured in that country, for that type of patient. For example, the third column is calculated by 294/1752*100.

^c^ This comprises *Aeromonas* spp. (n=1), *Citrobacter* spp. (n=16), *Enterobacter* spp. (n=24), *Moraxella* spp. (n=1), *Morganella* spp. (n=6), *Pantoea* spp. (n=2), *Providencia* spp. (n=2), *Salmonella* spp. (n=2), *Serratia* spp. (n=4), *Shigella* spp. (n=1), *Stenotrophomonas* spp. (n=1), and undetermined Gram-negative bacteria (n=115)

^d^ This comprises *Bacillus* spp. (n=19), *Clostridium* spp. (n=1), *Corynebacterium* spp. (n=1), *Lactobacillus* spp. (n=6), *Streptococcus* spp. (n=50), and undetermined Gram-positive bacteria (n=16).

**Table S5.** Antibiotic susceptibility, ESBL and MDR rates of *Enterobacterales*

|  | ***Escherichia coli*** | | | | | | | | ***Klebsiella* spp.** | | | | | | | | **Other *Enterobacterales*^a^** | | | | | | | |
| --- | --- | --- | --- | --- | --- | --- | --- | --- | --- | --- | --- | --- | --- | --- | --- | --- | --- | --- | --- | --- | --- | --- | --- | --- |
|  | **Kenya** | | **Tanzania** | | **Uganda** | | **Three countries** | | **Kenya** | | **Tanzania** | | **Uganda** | | **Three countries** | | **Kenya** | | **Tanzania** | | **Uganda** | | **Three countries** | |
|  | %^b^ | n^c^ | % | n | % | n | % | n | % | n | % | n | % | n | % | n | % | n | % | n | % | n | % | n |
| **Ampicillin** | 21.8 | 317 | 87.1 | 402 | 81.7 | 262 | 64.5 | 981 | NA | NA | 92.2 | 90 | 95.3 | 64 | 93.5 | 154 | 11.5 | 78 | 84.3 | 51 | 80.0 | 25 | 46.8 | 154 |
| **Amoxicillin- clavulanic acid** | 17.4 | 316 | 43.0 | 402 | 56.3 | 252 | 38.1 | 970 | NA | NA | 58.9 | 90 | 61.9 | 63 | 60.1 | 153 | 10.3 | 78 | 64.7 | 51 | 66.7 | 24 | 37.3 | 153 |
| **Ceftazidime** | 8.5 | 317 | 28.1 | 402 | 31.7 | 252 | 22.7 | 971 | NA | NA | 47.8 | 90 | 31.7 | 63 | 41.2 | 153 | 3.8 | 78 | 23.5 | 51 | 37.5 | 24 | 15.7 | 153 |
| **Ceftriaxone** | 9.2 | 315 | 32.1 | 402 | 46.2 | 262 | 28.5 | 979 | NA | NA | 52.2 | 90 | 50.0 | 64 | 51.3 | 154 | 5.1 | 78 | 25.5 | 51 | 56.0 | 25 | 20.1 | 154 |
| **Ciprofloxacin** | 30.0 | 317 | 57.0 | 402 | 47.9 | 263 | 45.8 | 982 | NA | NA | 55.6 | 90 | 26.6 | 64 | 43.5 | 154 | 33.8 | 77 | 45.1 | 51 | 44.0 | 25 | 39.2 | 153 |
| **Gentamicin** | 16.9 | 314 | 27.6 | 402 | 19.0 | 184 | 22.1 | 900 | NA | NA | 31.1 | 90 | 10.3 | 39 | 24.8 | 129 | 11.8 | 76 | 21.6 | 51 | 7.1 | 14 | 14.9 | 141 |
| **Nitrofurantoin** | 2.9 | 313 | 6.7 | 402 | 1.5 | 263 | 4.1 | 978 | NA | NA | 18.9 | 90 | 7.8 | 64 | 14.3 | 154 | 10.3 | 78 | 31.4 | 51 | 44.0 | 25 | 22.7 | 154 |
| **Trimethoprim** | 57.9 | 316 | 84.1 | 402 | 79.5 | 263 | 74.4 | 981 | NA | NA | 68.9 | 90 | 54.7 | 64 | 63.0 | 154 | 65.4 | 78 | 62.7 | 51 | 52.0 | 25 | 62.3 | 154 |
| **ESBL^d^** | 9.5 | 317 | 32.6 | 402 | 48.3 | 263 | 29.3 | 982 | NA | NA | 53.3 | 90 | 54.7 | 64 | 53.9 | 154 | 6.4 | 78 | 29.4 | 51 | 56.0 | 25 | 22.1 | 154 |
| **MDR^e^** | 22.7 | 317 | 66.4 | 402 | 66.2 | 263 | 52.2 | 982 | NA | NA | 62.2 | 90 | 34.4 | 64 | 50.6 | 154 | 19.2 | 78 | 43.1 | 51 | 44.0 | 25 | 31.2 | 154 |

^a^ Other *Enterobacterales* includes *Citrobacter*, *Enterobacter*, *Morganella*, *Proteus*, *Providencia*, *Pantoea*, *Salmonella*, *Serratia* and *Shigella* species.

^b^ % is the frequency of non-susceptible isolates, expressed in percentage.

^c^ n is the total number of isolates that were tested for a specific antibiotic.

^d^ Possible producers of ESBL were determined by considering the resistance to antibiotics Ceftazidime and Ceftriaxone, according to CLSI guidelines.^5^

^e^ Multi drug-resistance (MDR) was defined as non-susceptibility to at least one antimicrobial agent in three or more antimicrobial categories, according to the ECDC guidelines with some modifications, as described in the methods section. ^7^

**Table S6.** Antibiotic susceptibility, ESBL and MDR rates of relevant *Enterobacterales* isolated from outpatients with UTI.

|  | ***Escherichia coli* (outpatient)** | | | | | | | | ***Klebsiella* spp. (outpatient)** | | | | | | | | **Other *Enterobacterales*^a^ (outpatient)** | | | | | | | |
| --- | --- | --- | --- | --- | --- | --- | --- | --- | --- | --- | --- | --- | --- | --- | --- | --- | --- | --- | --- | --- | --- | --- | --- | --- |
| **Country** | **Kenya** | | **Tanzania** | | **Uganda** | | **Three**  **countries** | | **Kenya** | | **Tanzania** | | **Uganda** | | **Three**  **countries** | | **Kenya** | | **Tanzania** | | **Uganda** | | **Three**  **countries** | |
|  | **%^b^** | **N^c^** | **%** | **n** | **%** | **n** | **%** | **n** | **%** | **n** | **%** | **n** | **%** | **n** | **%** | **n** | **%** | **n** | **%** | **n** | **%** | **n** | **%** | **n** |
| **Ampicillin** | 21.1 | 294 | 85.7 | 350 | 81.6 | 261 | 63.5 | 905 | NA | NA | 93.8 | 64 | 95.2 | 63 | 94.5 | 127 | 11.0 | 73 | 82.5 | 40 | 80 | 25 | 44.2 | 138 |
| **Amoxicillin/**  **clavulanic acid** | 17.1 | 293 | 39.1 | 350 | 56.6 | 251 | 36.8 | 894 | NA | NA | 54.7 | 64 | 61.3 | 62 | 57.9 | 126 | 9.6 | 73 | 57.5 | 40 | 66.7 | 24 | 33.6 | 137 |
| **Ceftazidime** | 7.5 | 294 | 22.6 | 350 | 31.9 | 251 | 20.2 | 895 | NA | NA | 35.9 | 64 | 30.6 | 62 | 33.3 | 126 | 1.4 | 73 | 15.0 | 40 | 37.5 | 24 | 11.7 | 137 |
| **Ceftriaxone** | 8.2 | 293 | 26.0 | 350 | 46.0 | 261 | 26.0 | 904 | NA | NA | 42.2 | 64 | 49.2 | 63 | 45.7 | 127 | 2.7 | 73 | 17.5 | 40 | 56.0 | 25 | 16.7 | 138 |
| **Ciprofloxacin** | 29.6 | 294 | 54.0 | 350 | 47.7 | 262 | 44.3 | 906 | NA | NA | 50 | 64 | 25.4 | 63 | 37.8 | 127 | 31.9 | 72 | 42.5 | 40 | 44.0 | 25 | 37.2 | 137 |
| **Gentamicin** | 14.7 | 292 | 24.3 | 350 | 19.0 | 184 | 19.7 | 826 | NA | NA | 23.4 | 64 | 10.3 | 39 | 18.4 | 103 | 9.9 | 71 | 15.0 | 40 | 7.1 | 14 | 11.2 | 125 |
| **Nitrofurantoin** | 2.1 | 291 | 4.9 | 350 | 1.5 | 262 | 3.0 | 903 | NA | NA | 15.6 | 64 | 7.9 | 63 | 11.8 | 127 | 11.0 | 73 | 32.5 | 40 | 44.0 | 25 | 23.2 | 138 |
| **Trimethoprim** | 59.0 | 293 | 83.4 | 350 | 79.4 | 262 | 74.4 | 905 | NA | NA | 60.9 | 64 | 54.0 | 63 | 57.5 | 127 | 64.4 | 73 | 62.5 | 40 | 52.0 | 25 | 61.6 | 138 |
| **ESBL^d^** | 8.5 | 294 | 26.6 | 350 | 48.1 | 262 | 26.9 | 906 | NA | NA | 43.8 | 64 | 54.0 | 63 | 48.8 | 127 | 2.7 | 73 | 22.5 | 40 | 56.0 | 25 | 18.1 | 138 |
| **MDR^e^** | 21.4 | 294 | 64.0 | 350 | 66.0 | 262 | 50.8 | 906 | NA | NA | 56.3 | 64 | 33.3 | 63 | 44.9 | 127 | 16.4 | 73 | 40 | 40 | 44.0 | 25 | 28.3 | 138 |

^a^ Other *Enterobacterales* includes *Citrobacter*, *Enterobacter*, *Morganella*, *Proteus*, *Providencia*, *Pantoea*, *Salmonella*, *Serratia* and *Shigella* species.

^b^ % is the frequency of non-susceptible isolates, expressed in percentage.

^c^ n is the total number of isolates that were tested for a specific antibiotic.

^d^ Possible producers of ESBL were determined by considering the resistance to antibiotics Ceftazidime and Ceftriaxone, according to CLSI guidelines.^5^

^e^ Multi drug-resistance (MDR) was defined as non-susceptibility to at least one antimicrobial agent in three or more antimicrobial categories, according to the ECDC guidelines.^7^

**Table S7.** Antibiotic susceptibility, ESBL and MDR rates of relevant *Enterobacterales* isolated from inpatients with UTI.

|  | ***Escherichia coli* (inpatient)** | | | | | | | | ***Klebsiella* spp. (inpatient)** | | | | | | | | **Other *Enterobacterales*^a^ (inpatient)** | | | | | |
| --- | --- | --- | --- | --- | --- | --- | --- | --- | --- | --- | --- | --- | --- | --- | --- | --- | --- | --- | --- | --- | --- | --- |
| **Country** | **Kenya** | | **Tanzania** | | **Uganda** | | **Three**  **countries** | | **Kenya** | | **Tanzania** | | **Uganda** | | **Three**  **countries** | | **Kenya** | | **Tanzania** | | **Three**  **Countries^b^** | |
|  | **%^c^** | **n^d^** | **%** | **n** | **%** | **n** | **%** | **n** | **%** | **n** | **%** | **n** | **%** | **n** | **%** | **n** | **%** | **n** | **%** | **n** | **%** | **n** |
| **Ampicillin** | 30.4 | 23 | 96.2 | 52 | 100 | 1 | 76.3 | 76 | NA | NA | 88.5 | 26 | 100 | 1 | 88.9 | 27 | 20 | 5 | 90.9 | 11 | 68.8 | 16 |
| **Amoxicillin/**  **clavulanic acid** | 21.7 | 23 | 69.2 | 52 | 0 | 1 | 53.9 | 76 | NA | NA | 69.2 | 26 | 100 | 1 | 70.4 | 27 | 20 | 5 | 90.9 | 11 | 68.8 | 16 |
| **Ceftazidime** | 21.7 | 23 | 65.4 | 52 | 0 | 1 | 51.3 | 76 | NA | NA | 76.9 | 26 | 100 | 1 | 77.8 | 27 | 40 | 5 | 54.5 | 11 | 50 | 16 |
| **Ceftriaxone** | 22.7 | 22 | 73.1 | 52 | 100 | 1 | 58.7 | 75 | NA | NA | 76.9 | 26 | 100 | 1 | 77.8 | 27 | 40 | 5 | 54.5 | 11 | 50 | 16 |
| **Ciprofloxacin** | 34.8 | 23 | 76.9 | 52 | 100 | 1 | 64.5 | 76 | NA | NA | 69.2 | 26 | 100 | 1 | 70.4 | 27 | 60 | 5 | 54.5 | 11 | 56.3 | 16 |
| **Gentamicin** | 45.5 | 22 | 50 | 52 | NA^e^ | NA | 48.6 | 74 | NA | NA | 50 | 26 | NA | NA | 50.0 | 26 | 40 | 5 | 45.5 | 11 | 43.8 | 16 |
| **Nitrofurantoin** | 13.6 | 22 | 19.2 | 52 | 0 | 1 | 17.3 | 75 | NA | NA | 26.9 | 26 | 0 | 1 | 25.9 | 27 | 0 | 5 | 27.3 | 11 | 18.8 | 16 |
| **Trimethoprim** | 43.5 | 23 | 88.5 | 52 | 100 | 1 | 75.0 | 76 | NA | NA | 88.5 | 26 | 100 | 1 | 88.9 | 27 | 80 | 5 | 63.6 | 11 | 68.8 | 16 |
| **ESBL^f^** | 21.7 | 23 | 73.1 | 52 | 100 | 1 | 57.9 | 76 | NA | NA | 76.9 | 26 | 100 | 1 | 77.8 | 27 | 60 | 5 | 54.5 | 11 | 56.3 | 16 |
| **MDR^g^** | 39.1 | 23 | 82.7 | 52 | 100 | 1 | 69.7 | 76 | NA | NA | 76.9 | 26 | 100 | 1 | 77.8 | 27 | 60 | 5 | 54.5 | 11 | 56.3 | 16 |

^a^ Other *Enterobacterales* includes *Citrobacter*, *Enterobacter*, *Morganella*, *Proteus*, *Providencia*, *Pantoea*, *Salmonella*, *Serratia* and *Shigella* species.

^b^ No *Enterobacterales* were isolated from Uganda inpatients

^c^ % is the frequency of non-susceptible isolates, expressed in percentage.

^d^ n is the total number of isolates that were tested for a specific antibiotic.

^e^ NA: not applicable.

^f^ Possible producers of ESBL were determined by considering the resistance to antibiotics Ceftazidime and Ceftriaxone, according to CLSI guidelines.^5^

^g^ Multi drug-resistance (MDR) was defined as non-susceptibility to at least one antimicrobial agent in three or more antimicrobial categories, according to the ECDC guidelines with some modifications, as described in the methods section.^7^

**Table S8.** Antibiotic susceptibility and MDR rates of relevant Gram-positive uropathogens**.**

|  | ***Staphylococcus* spp.** | | | | | | | | ***Enterococcus* spp.** | | | | | | | |
| --- | --- | --- | --- | --- | --- | --- | --- | --- | --- | --- | --- | --- | --- | --- | --- | --- |
|  | **Kenya** | | **Tanzania** | | **Uganda** | | **Three countries** | | **Kenya** | | **Tanzania** | | **Uganda** | | **Three countries** | |
|  | **%^a^** | **n^b^** | **%** | **n** | **%** | **n** | **%** | **n** | **%** | **n** | **%** | **n** | **%** | **n** | **%** | **n** |
| **Cefoxitin** | 37.5 | 384 | 42.4 | 217 | 42.9 | 91 | 39.7 | 692 | NA | NA | NA | NA | NA | NA | NA | NA |
| **Ciprofloxacin** | 29.4 | 385 | 50.9 | 220 | 45.1 | 91 | 38.2 | 696 | 33.7 | 86 | 48.3 | 58 | 66.7 | 3 | 40.1 | 147 |
| **Nitrofurantoin** | 5.5 | 381 | 6.4 | 220 | 3.3 | 91 | 5.5 | 692 | 4.7 | 86 | 15.5 | 58 | 33.3 | 3 | 9.5 | 147 |
| **Trimethoprim** | 78.4 | 385 | 85.0 | 220 | 87.9 | 91 | 81.8 | 696 | NA | NA | NA | NA | NA | NA | NA | NA |
| **Gentamicin** | 18.2 | 385 | 24.1 | 220 | 26.6 | 64 | 20.9 | 669 | NA | NA | NA | NA | NA | NA | NA | NA |
| **Linezolid** | 23.4 | 376 | 5.5 | 220 | 7.7 | 65 | 15.9 | 661 | 14.6 | 82 | 0.0 | 52 | 0.0 | 2 | 8.8 | 136 |
| **Erythromycin** | 68.7 | 377 | 79.1 | 220 | 72.3 | 65 | 72.5 | 662 | 67.1 | 82 | 74.5 | 55 | 50.0 | 2 | 69.8 | 139 |
| **Tetracycline** | 41.5 | 386 | 53.6 | 220 | 67.7 | 65 | 48.0 | 671 | 37.6 | 85 | 69.0 | 58 | 50.0 | 2 | 50.3 | 145 |
| **Vancomycin** | NA | NA | NA | NA | NA | NA | NA | NA | 42.5 | 80 | 30.9 | 55 | 0.0 | 2 | 37.2 | 137 |
| **MDR^c^** | 54.7 | 386 | 72.3 | 220 | 54.9 | 91 | 60.3 | 697 | 32.6 | 86 | 46.6 | 58 | 33.3 | 3 | 38.1 | 147 |

^a^ % is the frequency of non-susceptible isolates, expressed in percentage.

^b^ n is the total number of isolates that were tested for a specific antibiotic.

^c^ Multi drug-resistance (MDR) was defined as non-susceptibility to at least one antimicrobial agent in three or more antimicrobial categories, according to the ECDC guidelines with some modifications, as described in the methods section.^7^

**Table S9.** Antibiotic susceptibility and MDR rates of *Staphylococcus* spp. and *Enterococcus* spp. isolated from outpatients with UTI.

|  | ***Staphylococcus* spp. (outpatient)** | | | | | | | | ***Enterococcus* spp. (outpatient)** | | | | | | | |
| --- | --- | --- | --- | --- | --- | --- | --- | --- | --- | --- | --- | --- | --- | --- | --- | --- |
| **Country** | **Kenya** | | **Tanzania** | | **Uganda** | | **Three countries** | | **Kenya** | | **Tanzania** | | **Uganda** | | **Three countries** | |
|  | **%^a^** | **n^b^** | **%** | **n** | **%** | **n** | **%** | **n** | **%** | **n** | **%** | **n** | **%** | **n** | **%** | **n** |
| **Cefoxitin** | 35.1 | 356 | 43.1 | 202 | 42.9 | 91 | 38.7 | 649 | NA**^c^** | NA | NA | NA | NA | NA | NA | NA |
| **Ciprofloxacin** | 28.3 | 357 | 50.7 | 205 | 45.1 | 91 | 37.7 | 653 | 32.1 | 81 | 42.6 | 47 | 66.7 | 3 | 36.6 | 131 |
| **Nitrofurantoin** | 4.5 | 353 | 5.9 | 205 | 3.3 | 91 | 4.8 | 649 | 4.9 | 81 | 19.1 | 47 | 33.3 | 3 | 10.7 | 131 |
| **Trimethoprim** | 77.9 | 357 | 85.4 | 205 | 87.9 | 91 | 81.6 | 653 | NA | NA | NA | NA | NA | NA | NA | NA |
| **Gentamicin** | 15.1 | 357 | 23.4 | 205 | 26.6 | 64 | 19.0 | 626 | NA | NA | NA | NA | NA | NA | NA | NA |
| **Linezolid** | 20.9 | 349 | 4.9 | 205 | 7.7 | 65 | 14.2 | 619 | 15.6 | 77 | 0.0 | 41 | 0.0 | 2 | 10.0 | 120 |
| **Erythromycin** | 67.4 | 350 | 78.0 | 205 | 72.3 | 65 | 71.5 | 620 | 64.9 | 77 | 70.5 | 44 | 50.0 | 2 | 66.7 | 123 |
| **Tetracycline** | 40.8 | 358 | 53.2 | 205 | 67.7 | 65 | 47.6 | 628 | 35.0 | 80 | 63.8 | 47 | 50.0 | 2 | 45.7 | 129 |
| **Vancomycin** | NA | NA | NA | NA | NA | NA | NA | NA | 41.3 | 75 | 29.5 | 44 | 0.0 | 2 | 36.4 | 121 |
| **MDR^d^** | 53.1 | 358 | 72.7 | 205 | 54.9 | 91 | 59.5 | 654 | 30.9 | 81 | 40.4 | 47 | 33.3 | 3 | 34.4 | 131 |

^a^ % is the frequency of non-susceptible isolates, expressed in percentage.

^b^ n is the total number of isolates that were tested for a specific antibiotic.

^c^ NA: not applicable.

^d^ Multi drug-resistance (MDR) was defined as non-susceptibility to at least one antimicrobial agent in three or more antimicrobial categories, according to the ECDC guidelines with some modifications, as described in the methods section.^7^

**Table S10.** Antibiotic susceptibility and MDR rates of *Staphylococcus* spp. and *Enterococcus* spp. isolated from inpatients with UTI.

|  | ***Staphylococcus* spp. (inpatient)** | | | | | | ***Enterococcus* spp. (inpatient)** | | | | | |
| --- | --- | --- | --- | --- | --- | --- | --- | --- | --- | --- | --- | --- |
| **Country** | **Kenya** | | **Tanzania** | | **Three countries^a^** | | **Kenya** | | **Tanzania** | | **Three countries^a^** | |
|  | **%^b^** | **n^c^** | **%** | **n** | **%** | **n** | **%** | **n** | **%** | **n** | **%** | **n** |
| **Cefoxitin** | 67.9 | 28 | 33.3 | 15 | 55.8 | 43 | NA^d^ | NA | NA | NA | NA | NA |
| **Ciprofloxacin** | 42.9 | 28 | 53.3 | 15 | 46.5 | 43 | 60.0 | 5 | 72.7 | 11 | 68.8 | 16 |
| **Nitrofurantoin** | 17.9 | 28 | 13.3 | 15 | 16.3 | 43 | 0.0 | 5 | 0.0 | 11 | 0.0 | 16 |
| **Trimethoprim** | 85.7 | 28 | 80.0 | 15 | 83.7 | 43 | NA | NA | NA | NA | NA | NA |
| **Gentamicin** | 57.1 | 28 | 33.3 | 15 | 48.8 | 43 | NA | NA | NA | NA | NA | NA |
| **Linezolid** | 55.6 | 27 | 13.3 | 15 | 40.5 | 42 | 0.0 | 5 | 0.0 | 11 | 0.0 | 16 |
| **Erythromycin** | 85.2 | 27 | 93.3 | 15 | 88.1 | 42 | 100 | 5 | 90.9 | 11 | 93.8 | 16 |
| **Tetracycline** | 50.0 | 28 | 60.0 | 15 | 53.5 | 43 | 80.0 | 5 | 90.9 | 11 | 87.5 | 16 |
| **Vancomycin** | NA | NA | NA | NA | NA | NA | 60.0 | 5 | 36.4 | 11 | 43.8 | 16 |
| **MDR^e^** | 75.0 | 28 | 66.7 | 15 | 72.1 | 43 | 60.0 | 5 | 72.7 | 11 | 68.8 | 16 |

^a^ No *Staphylococcus* or *Enterococcus* were isolated from Uganda inpatients

^b^% is the frequency of non-susceptible isolates, expressed in percentage.

^c^ n is the total number of isolates that were tested for a specific antibiotic.

^d^ NA: not applicable.

^e^ Multi drug-resistance (MDR) was defined as non-susceptibility to at least one antimicrobial agent in three or more antimicrobial categories, according to the ECDC guidelines with some modifications, as described in the methods section.^7^

**References**

1. Geerlings SE. Clinical Presentations and Epidemiology of Urinary Tract Infections. *Microbiol Spectr* 2016; **4:** 10.1128/microbiolspec.UTI-0002-2012. https://doi.org/10.1128/microbiolspec.UTI-0002-2012.
2. Bonkat G, Pickard R, Bartoletti R et al. EAU guidelines on urological infections*. European Association of Urology* 2017; **18:** 22-6
3. Orenstein R, Wong ES. Urinary tract infections in adults. *Am Fam Physician* 1999; **59:** 1225-1237.
4. Ginsburg CM, McCracken GH Jr. Urinary tract infections in young infants. *Pediatrics* 1982; **69:** 409-12.
5. CLSI. Performance Standards for Antimicrobial Susceptibility Testing— Thirty-First Edition: M100. 2021.
6. EUCAST. The European Committee on Antimicrobial Susceptibility Testing. Breakpoint tables for interpretation of MICs and zone diameters. Version 11.0. 2021.
7. Magiorakos AP, Srinivasan A, Carey RB et al. Multidrug-resistant, extensively drug-resistant and pandrug-resistant bacteria: an international expert proposal for interim standard definitions for acquired resistance. *Clin Microbiol Infect* 2012; **18:** 268-81. <https://doi.org/10.1111/j.1469-0691.2011.03570.x>.
